# Anomaly Detection Using Signal Segmentation and One-Class Classification in Diffusion Process of Semiconductor Manufacturing

**DOI:** 10.3390/s21113880

**Published:** 2021-06-04

**Authors:** Kyuchang Chang, Youngji Yoo, Jun-Geol Baek

**Affiliations:** 1 Department of Industrial and Management Engineering, Korea University, Seoul 02841, Korea; kyuchang@korea.ac.kr; 2 Samsung Electronics Co., Ltd., Hwaseong-si 18448, Korea; yz.yoo@samsung.com

**Keywords:** anomaly detection, fault detection and classification (FDC), signal segmentation, one-class classification (OCC), local outlier factor (LOF), isolation forest (*iF*)

## Abstract

This paper proposes a new diagnostic method for sensor signals collected during semiconductor manufacturing. These signals provide important information for predicting the quality and yield of the finished product. Much of the data gathered during this process is time series data for fault detection and classification (FDC) in real time. This means that time series classification (TSC) must be performed during fabrication. With advances in semiconductor manufacturing, the distinction between normal and abnormal data has become increasingly significant as new challenges arise in their identification. One challenge is that an extremely high FDC performance is required, which directly impacts productivity and yield. However, general classification algorithms can have difficulty separating normal and abnormal data because of subtle differences. Another challenge is that the frequency of abnormal data is remarkably low. Hence, engineers can use only normal data to develop their models. This study presents a method that overcomes these problems and improves the FDC performance; it consists of two phases. Phase I has three steps: signal segmentation, feature extraction based on local outlier factors (LOF), and one-class classification (OCC) modeling using the isolation forest (*iF*) algorithm. Phase II, the test stage, consists of three steps: signal segmentation, feature extraction, and anomaly detection. The performance of the proposed method is superior to that of other baseline methods.

## 1. Introduction

Recent developments in smart manufacturing have significantly improved the quality of the equipment and process control. Semiconductor fabrication is a complex technological process involving hundreds of steps. Its equipment is becoming more automated, accurate, and efficient, making the detection of potential faults increasingly difficult [1]. High-performance process monitoring and profile analysis enable engineers to detect various abnormal events at an early stage and prevent faults from occurring downstream. During the fabrication process, a fault detection and classification (FDC) system is commonly used to detect faults in semiconductor manufacturing. An FDC identifies the effects of a potential fault on the observed variables and focuses on the process variables that are most relevant to diagnosis. Early and accurate anomaly detection provides engineers with more time to avoid severe equipment malfunctions. It also reduces downtime, improves production quality, and reduces manufacturing costs. Accurate and precise diagnosis of faults during manufacturing is essential to ensure efficient operation and reduce yield loss while effectively controlling the process. Advanced sensors collect large amounts of data, such as temperature, pressure, flow, and power; most of this information is time series (TS) data collected in real time. Performing FDCs in real time requires performing time series classification (TSC) during fabrication.

The aim of this study was to analyze the diffusion process in semiconductor manufacturing, and raw trace data were collected from an existing production line. The characteristics of our data present the following three challenges.

First, an extremely high FDC performance is required because faults directly impact productivity and yield. Nevertheless, some normal and abnormal data show only subtle differences, and general classification algorithms may have limitations in achieving high performance. Conventional FDC methods extract statistical summary features, such as the mean and variance from the trace signal, and use the features as input arguments for the classifiers. Such statistical features have the limitation that a signal’s pattern information may be lost during summarization. Furthermore, the loss of timing information that the signal previously kept has a negative effect on the cause analysis to be performed later.

Second, a critical problem is that the frequency of abnormal data is remarkably low; therefore, the number of data that engineers can use to develop a model is limited. In this case, standard classification algorithms create a serious type II error that misclassifies faulty data as normal because the determination of FDC model parameters is biased to the majority class. Although various TSC methods have been verified for their performance, such as the 1- nearest neighbor (NN), dynamic time warping (DTW) [2], bag-of-SFA-symbols (BOSS) [3], shapelet transform [4], and hierarchical vote collective of transformation-based ensembles (HIVE-COTE) [5], it is difficult to apply current TSC methods to class imbalances. Representative algorithms that can be applied to the class imbalance problem are the local outlier factor (LOF) [6], isolation forest (*iF*) [7], one-class SVM [8], T^2^ control chart [9], and one-class incremental clustering [10]. However, the above methods for the class imbalance problem are not guaranteed to provide high performance when trained with raw trace data. Useful summary features must be extracted using an applicable process.

Finally, sensor noise is another factor that degrades the distinction between normal and abnormal trace data. Sensor noise is mainly caused by aging of the sensor and contamination of the processing facility. This noise is particularly severe when the environment or process sequence change, which degrades the performance of general FDC models without preprocessing. It is essential to develop a robust methodology to overcome sensor noise.

Various studies have been conducted to solve the above problems, especially FDC research and one-class time series classification (OCTSC) research, which are representative. Concerning the FDC domain, the standard literature addresses the use of full trace data to determine wafer faults. Chien et al. [1] decomposed the distribution of chemical vapor decomposition (CVD) trace data into a score distribution in a principal component (PC) space and a residual distribution using multiway principal component analysis (MPCA) [11]. The authors used the D and Q statistics of the two distributions as fault-monitoring indices. The D statistic is the Mahalanobis distance between the new wafer and the normal condition in a score space formed by fewer PCs. The Q statistic involves information on the variation. He and Wang [12] proposed a one-class k-nearest neighbor (kNN) method that calculates the distance from neighboring normal wafers and considers faults as outliers in the distribution. Subsequently, the authors used the kNN method on a PC space created by the MPCA [13]. As deep learning has made tremendous progress in recent years, Lee et al. [14] proposed a convolutional neural network model that detects CVD process faults and finds fault-introducing process parameters.

With regard to the OCTSC field, a recent method using dissimilarity-based representations (DBR) showed satisfactory performance. Mauceri et al. [14] used one-class nearest-neighbor classifiers on the DBR and proved that the classification results are more competitive overall than those from robust baseline algorithms. Both recent FDC and OCTSC algorithms are competitive in certain situations, but they are not comprehensively optimized for the three problems described above. We present signal segmentation and a one-class classification (OCC)-based FDC solution that overcomes the problems described above. The contributions of this study are as follows:

- The proposed method outperformed the existing methods and had the highest accuracy and F1-score.
- Because modeling is possible under extreme conditions of class imbalance, it can be applied to various manufacturing fields.

The remainder of this paper is organized as follows: Section 2 explains the fundamental problems, showing an example with real data; Section 3 introduces the proposed method in detail; Section 4 describes the experimental results to demonstrate the efficiency of the proposed methodology; and Section 5 presents the conclusions and recommendations for future studies.

## 2. Application Problem

This study was conducted using data from the diffusion process used in semiconductor manufacturing. The data collected from the sensors are a collection of values sequentially ordered over time. Each dataset is a collection of data from the same process using the same procedure. In other words, the 18 datasets used in the experiment are data collected in different process environments, and our experiments can cover various situations. The data used in this study have the following characteristics.

First, each of the 18 datasets had several change points as the step of the process changed over time. Whether a process step has changed can be estimated relatively clearly by checking if a mean shift has occurred. The step change occurs simultaneously in normal and abnormal data. Most of the state-of-the-art manufacturing processes consist of multiple steps over a long period of time; thus, it is expected that their characteristics are similar to the data used in this study.

**Figure 1:**
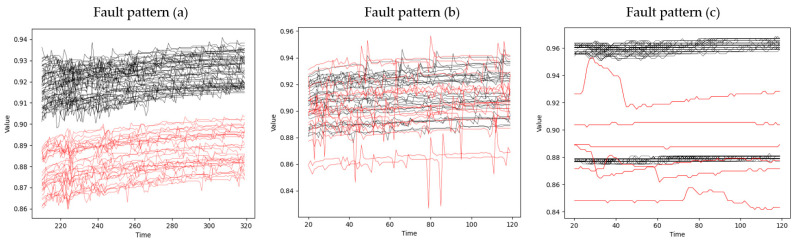
Three representative fault patterns.

Second, each signal has various types of fault patterns. The fault pattern refers to a peculiar pattern that is different from the normal signal appearing in abnormal data. To help understand the characteristics of the data used in this study, we defined some typical fault patterns in Figure 1. The normal signal is shown in black, and the corresponding fault pattern is shown in red. Fault pattern (a) is part of the signal where mean shift occurred. The mean shift occurring within an abnormal signal is more severe than that occurring within a normal signal. Fault pattern (b) is a part of the signal with a large variation. The degree of variation of the abnormal signal was greater than that of the normal signal. Fault pattern (c) is a part of the signal that has a different shape compared to the normal signal. The three patterns defined above appear randomly at specific steps in a specific process. In some steps, two or more patterns overlap.

**Figure 2:**
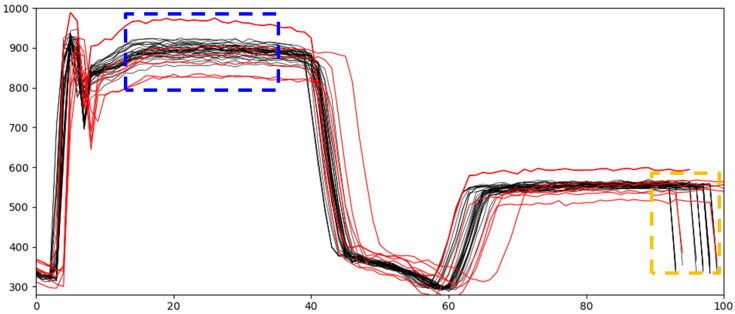
Example of normal (black) and abnormal (red) signals.

In addition to the above two characteristics, the frequency of abnormal signals is remarkably low, and sensor noise appears over several sections. The shape of the raw data and process information for each dataset cannot be disclosed because of the security guidelines of the data provider. Considering security, this section explains the motivation for our methodology through publicly available data that have a form similar to actual data. The data in this section represent the engineering variable from a LAM 9600 metal etcher used for etching wafers [11]. The semiconductor manufacturing process generates a large amount of data from the sensors belonging to the process equipment. A basic statistical process control (SPC) identifies faults from individual sensors to monitor the processing status, and the data in this section represent one of the sensor variables from a transformer-coupled plasma (TCP) metal etching process in a real semiconductor manufacturing process. This profile plot is similar to that generated by the sensor for process control. The data were obtained for 30 normal wafers and 10 wafers with intentionally induced faults. Figure 2 shows the plotted values over time from one sensor of the LAM 9600 metal etcher. The X-axis represents the process time, and the Y-axis indicates the radio frequency (RF) power value. The RF power value is an important sensor variable for monitoring the processing status of semiconductor manufacturing. The black lines represent normal data and the red lines indicate abnormal data. As shown in the orange dashed box, the region where data shifted along the X-axis is a noisy section, even within normal data. As shown in the blue dashed box, normal data are relatively clustered in the non-noisy section; data showing differences from clustered normal data are abnormal data. A more rigorous criterion for noise is presented in Section 3.1.

The data we used in this study were more difficult to deal with than the example data in Figure 2. Examining the actual data, we noted that the differences between the normal and abnormal data appeared only in a particular region. The noise level also varies with the signal region, and the number of data shifts is higher than that of the example data. A closer look at the two types of data in Figure 2 reveals that abnormal data are shifted in a particular region. The variation in a specific part of the signal was greater than that of the normal data. Because normal data and abnormal data overlap in many areas, it is difficult to distinguish and separate normal and abnormal datasets. Therefore, a new technique to improve the classification performance is needed.

## 3. Proposed Method

**Figure 3:**
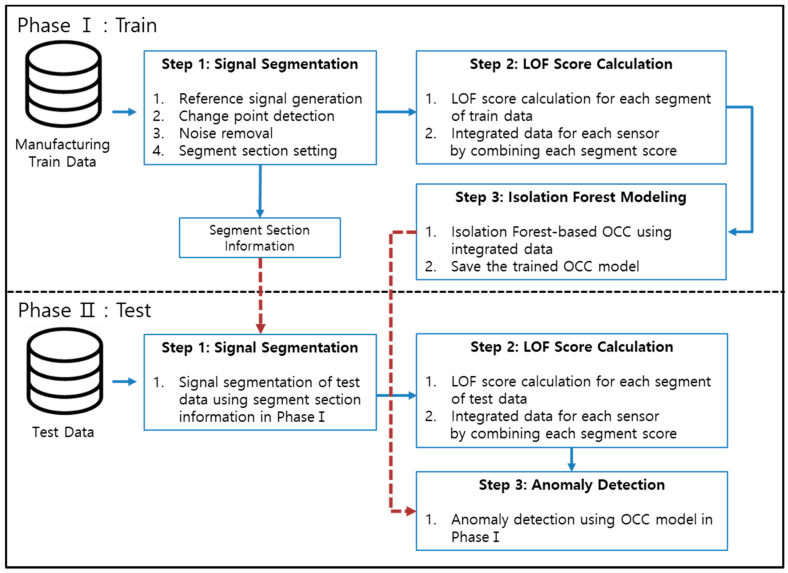
Schematic diagram of the proposed method.

This paper presents signal segmentation and an OCC-based anomaly detection technique specializing in signal data from the diffusion process in semiconductor manufacturing. The entire framework consists of two phases, one for training and the other for testing. This section explains the concept and application methods of each phase. Figure 3 shows a schematic of the proposed method.

Phase I consists of three steps for training the OCC model. Likewise, Phase II has three steps to detect anomalies with the OCC model trained in Phase I. Section 3.1 explains the process of signal segmentation in Phase I. Section 3.2, which accounts for the LOF-based anomaly score in Phase I. Section 3.3 describes the OCC modeling based on the isolation forest (*iF*) algorithm used in Phase I. The procedures for Phase II with test data are covered in Section 3.4. In addition, a rough concept of causal analysis is introduced in the same section. Hereafter “signal segment” refers to the part of a signal generated after signal segmentation. The signal segment has a value equal to the length of the time axis of the signal. In addition, this paper refers to a signal segment as a “data object” or simply “object.”

### 3.1. Phase I—Step 1: Signal Segmentation

In this section, we describe the signal segmentation process. We generate a reference signal that represents all the data collected from one sensor during the process using the same recipe. The reference signal is the average value at each time point of the normal signal. If the engineer agrees, a representative sample of normal data may be designated as the reference signal. To conduct signal segmentation, it is essential to detect the change point of the signal. Change-point detection (CPD), an important activity in statistics or signal processing, determines changes in the underlying model. We employ an offline detection framework as the reference signal is already given. Because the reference signal we created is a value measured over time, we assume that it is $y=\left\{y_{1},\ldots ,y_{T}\right\}$ [15]. The subscript of y refers to the time over which a signal of length *T* is sampled. The (b−a)- long sample subsignal ${\left\{y_{t}\right\}}_{t=a}^{b}\;(0\leq a<b\leq T)$ is denoted as $y_{a..b};$ the complete signal is therefore $y=y_{0..T.}$ In a strict sense, ${\left\{y_{t}\right\}}_{t=a}^{b}\;\mathrm{or}\;y_{a..b}$ is a value greater than a or equal to or less than b. It is assumed that the signal changes abruptly at unknown instants $t_{1}^{*}<\ldots <t_{i}^{*}<\ldots <t_{I}^{*}$. CPD consists of estimating the indices $t_{i}^{*}$. Formally, CPD is cast as a model selection problem, which consists of choosing the best possible segmentation according to a quantitative criterion V($\left\{t_{1},\;\ldots ,t_{I}\right\}$, y) that must be minimized. The criterion function V($\left\{t_{1},\;\ldots ,t_{I}\right\}$, y) for a particular segmentation is the sum of the costs of all segments that define the segmentation.

(1) $$V(\left\{t_{1},\;\ldots ,t_{I}\right\},\;y)\;:=\sum_{i=0}^{I}c\left(y_{t_{i}..t_{i+1}}\right)$$

where the symbol := means “is defined to be”. c(·) denotes the cost function that measures the goodness-of-fit of the subsignal $y_{t_{i}..t_{i+1}}$ to a specified model. The cost function is a measure of homogeneity. Intuitively, $c\left(y_{a..b}\right)$ is expected to be low if subsignal $y_{a..b}$ is homogeneous and large if the subsignal is heterogeneous. In this study, we used $C_{L_{2}}$ as the cost function for CPD [16]. $C_{L_{2}}$ is given by

(2) $$C_{L_{2}}\left(y_{a..b}\right)\;:=\sum_{t=a}^{b}\|y_{t}-{\bar{y}}_{a..b}{{\|}_{2}}^{2}$$

where ${\bar{y}}_{a..b}$ is the empirical mean of subsignal $y_{a..b}.$ It is assumed that the signal y is simply a sequence of independent normal random variables with a piecewise constant mean and the same variance. This cost function is referred to as the quadratic error loss and is known to be effective for mean-shift detection. We selected binary segmentation from various change-point searching methods because it yields an approximate solution [17]. Binary segmentation, denoted as BinSeg, is conceptually simple to implement. BinSeg is a greedy sequential algorithm, and the first change-point estimate ${\hat{t}}^{\left(1\right)}$ is given by

(3) $${\hat{t}}^{\left(1\right)}\;:=argmin_{1\leq t<T-1}c\left(y_{0..t}\right)+c\left(y_{t..T}\right)$$

**Figure 4:**
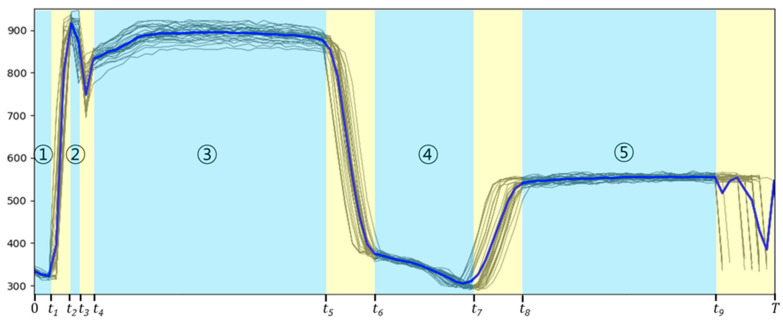
Example of signal segmentation.

This operation is performed greedily, in the sense that it searches for the change point that minimizes the sum of costs. The signal is then split into two at ${\hat{t}}^{\left(1\right)}$. The operation is repeated on the resulting subsignals until a stopping criterion is met. BinSeg is combined with $C_{L_{2}}$ and implemented to obtain candidates for change points by minimizing a cost function over possible change points. The stopping criterion determines the number of change points. The number of change points in the data is larger than the number of perceptible changes that the engineer can visually check. Figure 4 shows the results of applying the CPD algorithm to the sample data presented in Figure 2. The thick blue line represents the reference signal, which is the average value of the normal data. The black lines represent the normal data. In Figure 4, the detected change points are $\left\{t_{1},\ldots ,t_{9}\right\}$, dividing the signal into a total of 10 segments, $\left\{y_{0..t_{1}},\;y_{t_{2}..t_{3}\;},\ldots ,\;y_{t_{9}..T}\right\}.$ The area enclosed in the light yellow rectangle represents the noise. There are two criteria for determining noise based on an engineer’s knowledge. One is whether there is a significant difference in the data at the same point on the X-axis, and the other is the degree of data shift along the X-axis. Noise usually appears at the boundary of a segmented signal. When the signal is divided into a sufficiently large number of segments, noise appears as an independent segment with a short time interval so that it can be evaluated and removed easily. Noisy areas are excluded from the segments. The areas marked in light blue are signal segments that must be dealt with. Finally, five segments can be set based on the above criteria. The circled numbers in Figure 4 indicate the corresponding segment in the example data. For example, ① represents the first segment, and $y_{0..t_{1}}$ and ② represents the second segment, $y_{t_{1}..t_{2}}$. Segment information will be used in the remainder of the anomaly detection process.

### 3.2. Phase I—Step 2: LOF Score Calculation

The LOF-based anomaly score represents the degree of “outlierness” and is also called the LOF score or the LOF value in this study. In this section we describe how to obtain the LOF value for each section of the signal.

After splitting the signal into multiple segments, extracting quantitative measures for each segment is needed for training the FDC model. For example, because the signal in Figure 4 is divided into five sections, extracting five-dimensional multivariate information from the signal is the purpose of Step 2 in Phase I. This process is beneficial for reducing the number of dimensions and for the representation of raw data. One density-based outlier detection algorithm, LOF, has several advantages over other outlier detection algorithms [6,18]. Local outliers can be detected by providing the outlierness on a numerical scale on the degree of isolation of an object with respect to the surrounding neighborhood. As mentioned at the beginning of Section 3, the signal segment is also written as a data object and may have a sensor value equal to the time length of the signal. The Euclidian distance of the $k_{th}$ nearest object from an object *p* is computed and defined as the *k*–distance, where the user-defined parameter *k* is the number of nearest neighbors [19]. If the number of nearest neighbors is k, the algorithm is executed based on the following definitions: k–distance(p) is the distance between the data object p and its $k_{th}$ nearest neighbor. Given the k–distance(p), the k–distance neighborhood of p contains every object whose distance from p is not greater than the k–distance. This is expressed as N(p,k). The $reach–dist_{k}\left(p,o\right)$ is the reachability distance of object p with respect to object o. Here, the o symbol refers to the data within the k–distance of p.

(4) $$reach–dist_{k}\left(p,o\right)=\max\left\{k–distance\left(o\right),\;d\left(p,o\right)\right\}$$

where d(p,o) is the Euclidean distance between p and o. If the Euclidean distance of two points is very small, the following steps will use k–distance(o) instead of d(p,o) to represent $\;reach–dist_{k}\left(p,o\right).$ $lrd_{k}\left(p\right)\;$ is the local reachability density of an object p which is calculated according to the following equation.

(5) $$lrd_{k}\left(p\right)={\left(\;\frac{1}{k}\sum_{o\in N_{\left(p,k\right)}}reach–dist_{k}\left(p,o\right)\right)}^{-1}$$

where N(p,k) is the set of k nearest neighbors of p. $lrd_{k}\left(p\right)$ is the average reachability density of the k nearest neighbors. Intuitively, the local reachability density of an object p is the inverse of the average reachability distance based on its $k_{th}$ nearest neighbor. It should be noted that if p is located in a dense area, the denominator of $lrd_{k}\left(p\right)$ becomes small, increasing $lrd_{k}\left(p\right)$. In contrast, if p is located in a sparse area, the denominator of $lrd_{k}\left(p\right)$ becomes large, resulting in a small $lrd_{k}\left(p\right)$. $LOF_{k}\left(p\right)$ is the LOF at point p. This factor is given by

(6) $$LOF_{k}\left(p\right)=\frac{1}{k}\sum_{o\in N_{\left(p,k\right)}}\frac{lrd_{k}\left(o\right)}{lrd_{k}\left(p\right)}$$

$LOF_{k}\left(p\right)$ is the average of the ratios between $lrd_{k}\left(p\right)$ and those of the k–nearest neighbors of p, and represents the degree of outlierness for p. If p is not an outlier, the LOF value is close to one because their densities are similar. If p is an outlier, the LOF value is greater than one because the relative density of p is smaller than N(p,k). This means that the object is located far from normal samples so that the LOF value increases as the degree of outlierness increases.

In this study, the LOF-based anomaly scores for each segment in each signal were calculated for anomaly detection. In Phase I, we obtained the score for the signal corresponding to each segment in the training set. We searched for a value of k between 15 and 25 and set it to 20, which shows high performance empirically. As a result, the LOF score quantifies the difference between the normal and abnormal signals. In the example data presented in Figure 2, we calculated the LOF scores for each of the five segments. In other words, the number of segments corresponds to the number of variables for one signal.

There are two advantages deriving from the score being calculated for each segment rather than for the entire signal. First, the data dimension was considerably reduced. The raw signal has as many variables as the number of time points, but in the method proposed in Section 3.2, each segment of the raw signal is compressed into one score. Second, owing to the LOF score for each segment, it was possible to monitor the sensor data for each section. If the LOF score of a specific segment is remarkably different from other scores in the same section, it can be inferred that there is a problem in that section.

### 3.3. Phase I—Step 3: Isolation Forest Modeling

The difference for each signal in each segment is quantified in Section 3.2. Using the quantified scores in the classification model is the next step. In this section, we use another OCC method. A one-class classifier aims to capture the characteristics of training instances to distinguish them from potential outliers. The *iF* method is an extension of decision trees based on isolation and is inspired by the random forest algorithm, and in several applications it has outperformed cutting-edge outlier detection [7]. The main idea of *iF* is that anomalies are far different from the rest of the data and are susceptible to isolation. Outliers can be divided from the remaining data through simple partitioning. The isolation procedure generates a tree with an observation at each leaf, and each internal node is associated with a split on one variable. The isolation procedure described above was repeated to generate different trees. The likelihood of an observation being an outlier is provided by a score. The score is correlated with the path lengths necessary to isolate that observation.

The proposed method includes *iF* modeling with LOF scores extracted from each signal in Section 3.2. Random partitioning can be represented by an ensemble of t binary trees. Anomalies produce mean paths from the root to leaves that are longer than those for normal attributes. Trees are called isolation trees (iTs). Given a dataset, each *iT* is obtained by selecting a random subset of attributes and dividing it by randomly selecting a feature and splitting the branch until the node has only one instance. The *iF* defines an anomaly score, which is a quantitative index that defines an outlier’s degree of isolation. The anomaly score is defined for an observation q as given in Equation (7). Observation q refers to the collection of the LOF scores of all segments for one signal. For example, because the signal in Figure 4 is divided into five segments, each observation comprises five-dimensional multivariate information, as explained in Section 3.2.

(7) $$S\left(x,\psi \right)=2^{\left(-\frac{E\left(h\left(q\right)\right)}{c\left(\psi \right)}\right)}\in \left[0,1\right]$$

where h(·) indicates the path length from a group of iT. That is, E(h(q)) is the average path length h(·) over the t iTs, and c(ψ)  is an adjustment factor used to normalize the path length,  ψ is the subsample size and the number of trees, and t needs to be sufficiently large to allow convergence of the path length. When E(h(q)) → *n* − 1, the anomaly score tends to zero, meaning that q appears to be a normal instance. On the other hand, when E(h(q)) → 0, the anomaly score tends to 1, meaning that p appears to be an outlier. The threshold of the *iF* model can be set by determining the number of outliers. In this study, we searched for the optimal outlier ratio; we tried the ratio values 0.005, 0.01, 0.15, and 0.2, and set it to 0.01, which had an excellent empirical performance. For all experimental data, the *iF* model threshold was set such that the outlier ratio was 0.01.

In this step, we train the *iF*-based anomaly detection model with LOF scores of signal segments extracted from the training set. We chose the *iF* for several reasons. First, it provides the best performance for the extracted features and is compatible with LOF-based features. Feature extraction solves the fundamental problem of *iF*, that is, the performance decreases on high-dimensional data. Second, *iF* is a representative OCC methodology that can be applied to class imbalance problems. *iF* is known for its low linear time complexity and memory requirements [7]. Third, because *iF* is a tree-based algorithm, it has the advantage of being able to calculate the causative factors. The process of calculating the causative segment after applying the *iF* algorithm is an important diagnostic procedure in the FDC domain.

### 3.4. Phase II

In Section 3, we present a methodology for training the OCC model. In this section, we present a procedure for testing in Phase II. It consists of three steps and has a structure similar to that in Phase I.

In Step 1 of Phase II, we conducted signal segmentation on the test data. In Section 3.1, segmentation using the reference data has already been performed, and we have the information of the segment section. Segment section information concludes the knowledge of the finally determined segments and the unnecessary sections, including sections with noise or no data. Based on this information, the test data are segmented.

In step 2 of Phase II, we obtained the LOF-based anomaly score between the signals corresponding to each segment in the test data. The method of extracting the LOF-based anomaly score was the same as that presented in Section 3.2. The only difference is that in this section the method was applied to test data, while previously it was applied to training data.

In step 3 of Phase II, we used the *iF*-based OCC model for anomaly detection, which was already trained in step 3 of Phase I. After applying the test data to the model, we made a final judgment on whether the data were abnormal.

## 4. Experiments

We performed the FDC task by sequentially applying the described procedures. In Section 4.1, we describe the data used in the experiments. In Section 4.2, we demonstrate the effectiveness of the proposed method by comparing it with other baseline OCC algorithms. For reference, the experiment was performed on a 3.60 GHz computer.

### 4.1. Data Description

This study was conducted using the data obtained from the diffusion process in actual semiconductor manufacturing. The diffusion process involves particle transfer from higher to lower regions of concentration [18]. When performing diffusion implantation, the key process introduces a controlled quantity of dopants into semiconductors to alter conductivity. Dopant atoms are introduced in the gas phase using doped oxide sources. Then, the doping concentration decreases monotonically from the surface, and several factors of the process determine the in-depth distribution of the dopant. Various types of trace data, including temperature, pressure, flow, and power, are generated during the process described above. The data gathered in the target process are TS data collected in real time.

We performed an analysis of 18 sets of sensor data. Each set was a collection of data from the same process. As explained in Section 2, the data collected from the sensors is a collection of values sequentially ordered in time and has the characteristics that are unique to semiconductor manufacturing. Because it is difficult to obtain fault data, hundreds of normal data are used in the training process. In each of the 18 datasets, about a hundred available abnormal data points were used as test data. We applied cross validation (CV) to validate the proposed method. We used 10-fold CV, a widely used validation method, to reduce the uncertainty. The 10-fold CV involves randomly dividing the set of observations into 10 folds of approximately equal size. Here, the set of observations refers to the training data. The first fold is left aside, and the method is performed on the remaining nine folds. Because the methodology we used in the experiment is an anomaly detection method based on OCC, the test set data is also divided into 10 folds. Likewise, the first fold is left aside, and the remaining nine folds are treated as a validation set. Subsequently, the accuracy and F1-score were computed. This procedure was repeated 10 times. When the number of training sets was more than three times the number of test sets, the number of normal and abnormal data was balanced by undersampling from the training set.

When performing 10-fold CV, the computation time was recorded while performing model training and testing, and the average time was recorded as the average calculation time of each algorithm. The computation time includes not only the time required to train and test the model, but also the time required for all preprocessing.

### 4.2. Comparison of Experimental Results with Other State-of-the-Art Methods

In Section 4.2, the performance of the proposed method is verified by comparing it with three basic OCC methods and state-of-the-art classification algorithms. Three basic OCC methods were selected because they were used as baselines in many OCC studies. We explained how to set the hyperparameter when training the LOF and *iF* algorithms in the proposed method in Section 3. The same criteria were applied to train the individual LOF and *iF* used in the comparison experiment. When training OC-SVM, we performed grid search to find out the optimal value of *Nu* and *Gamma*. *Nu*, the parameter that controls the training errors is set to 0.1, and *Gamma* which determines the influence of radius on the kernel is set to an optimal value for each experiment.

Only a few classification methods have recently been developed in the field of OCTSC. Among them, we compare experiments with algorithms that have already been proven to perform well. We constructed a comparison experiment with a one-class nearest-neighbor classifier using DBR. Mauceri et al. [14] evaluated various DBRs derived from dissimilarity and prototype methods. The DBR was obtained by calculating the dissimilarity between a single unknown object and a set of prototype objects. The prototype method determines how to extract a subset of prototypes from a set of training samples. In the experiment, when only 10–20% of the training samples were extracted as prototypes using the k–means algorithm, the classification performance was the best for dissimilarity measures. The centroids of the k clusters were used as the prototype objects. Therefore, we extracted some data as a prototype (10–20%) using the k–means algorithm, and calculated 10 dissimilarity measures. Specifically, the 10 dissimilarities were based on Kullback–Leibler [19], cosine dissimilarity [20], dynamic time warping (DTW) [21], autocorrelation [22], Chebyshev norms [23], Manhattan norms [24], Euclidean norms, Gaussian kernel [25], sigmoid kernel, and Wasserstein distance [26]. The 1NN classifier was used to classify the trace data. The results of the proposed algorithm are presented in the first column of the tables. The experimental results written in the “Raw” column of Table 1 and Table 2 mean that the raw data were utilized without calculating the dissimilarity measure. Next, basic OCC methodologies, including LOF, one-class SVM (OC-SVM), and isolation forest (*iF*), were arranged in order. The OCTSC results for the raw data and 10 dissimilarity measures were added to the tables. Table 1 summarizes the accuracy, and Table 2 summarizes the F1-score.

The average value in each column is shown, and the rank of the proposed algorithm among all comparison groups is presented in the last column. For each dataset, we calculated the rank of the proposed algorithm to compare it to the other methods.

As shown in Table 1, the proposed algorithm achieved the highest performance accuracy for almost all the datasets. As shown in Table 2, we can see that the proposed algorithm had the highest performance in the F1-score for almost all datasets.

**Figure 5:**
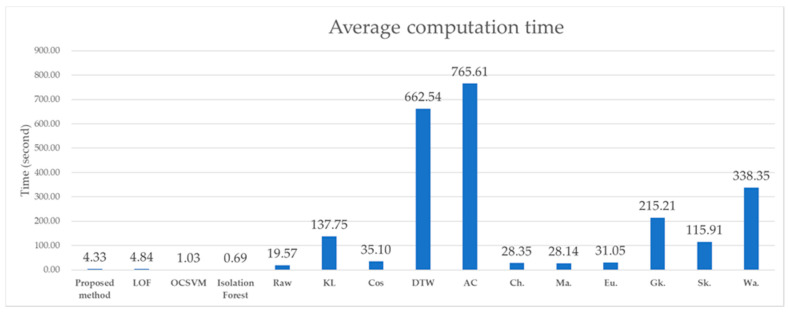
Average computation time. LOF: local outlier factor, OC-SVM: one-class support vector machine, *iF*: isolation forest, KL: Kullback-Leibler, Cos: cosine dissimilarity, DTW: dynamic time warping, AC: autocorrelation, Ch.: Chebyshev norms, Ma.: Manhattan norms, Eu.: Euclidean norms, Gk: Gaussian kernel, Sk.: Sigmoid kernel, Wa.: Wasserstein distance.

In terms of computation time, the proposed method exhibited excellent performance. Figure 5 shows the average value of the computation time for each algorithm as a bar graph. Many of the methods of Mauceri et al. [14] take significant time because they extract features based on the distance between data.

## 5. Conclusions

This study presented an advanced diagnostic methodology using signal segmentation and OCC in the diffusion process of semiconductor manufacturing. The entire framework consists of two phases, one for training and the other for testing. Each phase comprises three steps for anomaly detection.

By conducting signal segmentation, a method for utilizing signal data information was presented. Rather than using the raw signal, noise can be removed, and signal data can be precisely monitored for each section. The LOF-based score quantifies the difference between the normal and abnormal signals. Because normal signals are clustered with each other, and abnormal signals differ in density, LOF increases the distinction between a normal signal and an abnormal signal. The isolation forest (*iF)* technique was used as the final classification model, which is consistent with the previous steps.

The proposed method outperformed the existing methods, with the highest F1-score and accuracy score. Moreover, modeling is possible under extreme conditions of class imbalance. Thus, it can be applied to various manufacturing fields as well.

In the future, it will be necessary to conduct follow-up studies related to cause analysis, which are essential for manufacturing domains. Owing to the tree-based *iF* in the final step, a simple causal analysis is expected.

## Figures and Tables

**Table 1:** Experimental results in terms of accuracy.

	Proposed Method	LOF	OC- SVM	*iF*	Mauceri et al. [14]											Rank
					Raw	KL	Cos	DTW	AC	Ch.	Ma.	Eu.	Gk	Sk.	Wa.	
data#1	0.960	0.857	0.736	0.868	0.817	0.770	0.817	0.756	0.776	0.680	0.764	0.774	0.762	0.729	0.756	1
data#2	0.908	0.897	0.707	0.771	0.790	0.692	0.750	0.743	0.742	0.644	0.750	0.761	0.750	0.679	0.741	1
data#3	0.963	0.931	0.685	0.807	0.825	0.680	0.756	0.748	0.706	0.727	0.781	0.761	0.739	0.682	0.731	1
data#4	0.961	0.884	0.690	0.925	0.826	0.619	0.767	0.692	0.807	0.677	0.698	0.745	0.677	0.653	0.702	1
data#5	0.957	0.756	0.671	0.725	0.745	0.668	0.767	0.709	0.716	0.627	0.696	0.705	0.679	0.617	0.706	1
data#6	0.908	0.771	0.694	0.731	0.692	0.689	0.760	0.690	0.732	0.730	0.690	0.689	0.684	0.690	0.690	1
data#7	0.861	0.892	0.753	0.733	0.731	0.678	0.731	0.728	0.752	0.721	0.748	0.731	0.731	0.720	0.739	2
data#8	0.875	0.818	0.726	0.654	0.712	0.698	0.682	0.682	0.677	0.716	0.684	0.692	0.681	0.698	0.689	1
data#9	0.891	0.883	0.674	0.688	0.671	0.695	0.662	0.672	0.782	0.714	0.670	0.670	0.661	0.660	0.669	1
data#10	0.861	0.750	0.655	0.657	0.721	0.671	0.645	0.662	0.678	0.688	0.663	0.666	0.647	0.643	0.664	1
data#11	0.883	0.841	0.701	0.708	0.692	0.702	0.671	0.692	0.675	0.694	0.688	0.689	0.686	0.700	0.692	1
data#12	0.899	0.892	0.717	0.668	0.725	0.782	0.646	0.721	0.646	0.692	0.699	0.688	0.665	0.731	0.720	1
data#13	0.959	0.956	0.828	0.917	0.621	0.577	0.576	0.677	0.601	0.655	0.600	0.600	0.581	0.637	0.677	1
data#14	0.935	0.877	0.837	0.814	0.742	0.744	0.800	0.759	0.784	0.764	0.737	0.735	0.694	0.735	0.736	1
data#15	0.929	0.919	0.698	0.692	0.839	0.717	0.830	0.837	0.826	0.845	0.827	0.852	0.827	0.801	0.827	1
data#16	0.926	0.901	0.701	0.682	0.695	0.695	0.691	0.691	0.689	0.693	0.695	0.695	0.691	0.695	0.695	1
data#17	0.914	0.895	0.687	0.736	0.767	0.696	0.727	0.735	0.729	0.784	0.740	0.743	0.730	0.720	0.739	1
data#18	0.827	0.763	0.696	0.700	0.732	0.690	0.709	0.706	0.706	0.735	0.706	0.709	0.700	0.690	0.706	1
Avg.	0.912	0.860	0.714	0.749	0.741	0.693	0.721	0.717	0.724	0.710	0.713	0.717	0.699	0.693	0.716	1

LOF: local outlier factor, OC-SVM: one-class support vector machine, *iF*: isolation forest, KL: Kullback–Leibler, Cos: cosine dissimilarity, DTW: dynamic time warping, AC: autocorrelation, Ch.: Chebyshev norms, Ma.: Manhattan norms, Eu.: Euclidean norms, Gk: Gaussian kernel, Sk.: Sigmoid kernel, Wa.: Wasserstein distance.

**Table 2:** Experimental results in terms of F1-score.

	Proposed Method	LOF	OC- SVM	*iF*	Mauceri et al. [14]											Rank
					Raw	KL	Cos	DTW	AC	Ch.	Ma.	Eu.	Gk	Sk.	Wa.	
data#1	0.970	0.903	0.833	0.907	0.621	0.476	0.622	0.421	0.502	0.171	0.452	0.486	0.446	0.314	0.422	1
data#2	0.934	0.928	0.818	0.849	0.544	0.140	0.399	0.372	0.369	0.258	0.399	0.443	0.399	0.072	0.366	1
data#3	0.972	0.950	0.807	0.870	0.644	0.071	0.426	0.393	0.231	0.388	0.511	0.444	0.358	0.090	0.322	1
data#4	0.967	0.909	0.783	0.936	0.741	0.184	0.619	0.426	0.705	0.439	0.446	0.567	0.378	0.307	0.457	1
data#5	0.964	0.826	0.776	0.801	0.565	0.349	0.617	0.473	0.495	0.226	0.436	0.460	0.385	0.166	0.464	1
data#6	0.934	0.853	0.812	0.828	0.143	0.127	0.440	0.130	0.327	0.326	0.130	0.127	0.100	0.130	0.130	1
data#7	0.904	0.925	0.842	0.830	0.323	0.068	0.323	0.309	0.406	0.278	0.392	0.323	0.323	0.275	0.358	2
data#8	0.913	0.880	0.828	0.788	0.240	0.171	0.075	0.087	0.058	0.255	0.097	0.139	0.082	0.171	0.125	1
data#9	0.919	0.916	0.796	0.801	0.134	0.248	0.089	0.134	0.558	0.325	0.127	0.127	0.085	0.082	0.122	1
data#10	0.898	0.834	0.783	0.780	0.396	0.227	0.090	0.159	0.237	0.281	0.168	0.177	0.098	0.071	0.168	1
data#11	0.918	0.893	0.815	0.816	0.141	0.192	0.025	0.142	0.046	0.155	0.120	0.128	0.109	0.182	0.142	1
data#12	0.924	0.921	0.812	0.786	0.412	0.586	0.093	0.400	0.093	0.295	0.319	0.279	0.182	0.433	0.398	1
data#13	0.961	0.959	0.857	0.925	0.338	0.208	0.204	0.491	0.282	0.436	0.280	0.280	0.220	0.385	0.491	1
data#14	0.952	0.914	0.890	0.874	0.368	0.378	0.571	0.433	0.520	0.451	0.348	0.338	0.149	0.338	0.343	1
data#15	0.948	0.942	0.814	0.806	0.682	0.269	0.656	0.675	0.647	0.704	0.649	0.713	0.649	0.576	0.649	1
data#16	0.946	0.930	0.815	0.802	0.159	0.159	0.138	0.138	0.126	0.149	0.159	0.159	0.138	0.159	0.159	1
data#17	0.937	0.926	0.808	0.830	0.461	0.161	0.305	0.339	0.315	0.521	0.359	0.374	0.319	0.278	0.356	1
data#18	0.880	0.848	0.812	0.809	0.335	0.133	0.225	0.212	0.212	0.446	0.212	0.228	0.182	0.133	0.212	1
Avg.	0.936	0.903	0.817	0.835	0.403	0.230	0.329	0.318	0.341	0.339	0.311	0.322	0.256	0.231	0.316	1

LOF: local outlier factor, OC-SVM: one-class support vector machine, *iF*: isolation forest, KL: Kullback–Leibler, Cos: cosine dissimilarity, DTW: dynamic time warping, AC: autocorrelation, Ch.: Chebyshev norms, Ma.: Manhattan norms, Eu.: Euclidean norms, Gk.: Gaussian kernel, Sk.: Sigmoid kernel, Wa.: Wasserstein distance.

## Data Availability

Not applicable.
